# Association Between Lactate Trajectories and Clinical Outcomes in Critically Ill Patients with Sepsis-Associated Liver Injury

**DOI:** 10.3390/jcm15176837

**Published:** 2026-09-03

**Authors:** Hong Wei, Jian Zhang, Sijie Yu, Cheng Zhang, Yunlong Weng, Min Shao

**Affiliations:** 1 Department of Critical Care Medicine, The First Affiliated Hospital of Anhui Medical University, Hefei 230001, China; 2436020260@stu.ahmu.edu.cn (H.W.);; 2 Department of Emergency Medicine, Affiliated Lu’an Hospital of Anhui Medical University, Lu’an 237005, China; 3 Department of Anhui Provincial Cancer Institute, The First Affiliated Hospital of Anhui Medical University, Hefei 230022, China

**Keywords:** lactate trajectories, outcomes, sepsis-associated liver injury, critically ill

## Abstract

**Background**: Sepsis-associated liver injury (SALI) is a severe complication of sepsis that significantly increases the risk of mortality in intensive care unit (ICU) patients. While lactate is recognized as a prognostic biomarker for sepsis, the predictive potential of dynamic lactate trajectories in patients with SALI has not been investigated. This study aims to elucidate the association between serum lactate trajectories and clinical outcomes. **Methods**: Latent class trajectories of serum lactate dynamics were modeled in 524 patients with SALI from the MIMIC-IV dataset. Serum lactate levels were measured once daily for four consecutive days after ICU admission. Seven distinct trajectory phenotypes were identified, and their prognostic value was assessed using multivariate Cox regression. **Results**: The seven lactate trajectory phenotypes identified in SALI patients included the Class 1 low-stable, Class 2 moderate-slow decline, Class 3 low-slow decline, Class 4 moderate-slow decline and mild increase, Class 5 moderate-slow decline and rapid increase, Class 6 high-rapid decline, and Class 7 moderate-persistence groups. Fully adjusted multivariate Cox regression indicated significantly elevated mortality risk in Class 5 (HR 2.81, 95% CI 1.67–4.74) and Class 7 (HR 3.44, 95% CI 2.02–5.85) (all *p* < 0.001), whereas Classes 1–4 and 6 showed no significant differences. The XGBoost classifier achieved a test set AUROC of 0.7098 (training AUROC = 0.9029), indicating limited generalizability due to overfitting. SHAP analysis was used for exploratory purposes to identify potential predictors, with pH, platelets, and alkaline phosphatase emerging as dominant features. **Conclusions**: Substantial heterogeneity in lactate trajectories was found among patients with SALI, establishing these dynamic patterns as robust predictors of clinical outcomes.

## 1. Introduction

Sepsis is a syndrome caused by a dysregulated host response to infection, leading to life-threatening organ dysfunction. Sepsis affects millions of people each year worldwide [1,2]. The mortality rate for hospitalized patients with sepsis is approximately 35%, while that for those in the intensive care unit (ICU) is 41.9% [3,4]. Sepsis frequently leads to multiple organ dysfunction [5], with the liver being the organ directly involved in immune regulation and inflammatory responses, commonly exhibiting acute injury [6,7]. The pathogenesis of this liver injury has been linked to oxidative stress [8], the gut microbiota [9], endothelial damage [10] and inflammatory processes [11]. Recent studies have shown that sepsis-associated liver damage is an independent risk factor for multiple organ dysfunction and death in sepsis patients [12]. Therefore, the early identification and appropriate management of sepsis-associated liver injury (SALI) are of critical importance in preventing mortality.

Lactate is produced by the anaerobic glycolysis of glucose, primarily in skeletal muscles, the intestine, brain, skin, and red blood cells. The lactate produced by these cells enters the hepatic and renal circulation before undergoing oxidative metabolism. The liver clears between 70 and 80% of lactate, while the kidneys handle 20–30% [13,14]. Consequently, impaired liver function leads to elevated lactate levels, inducing conditions such as lactic acidosis. Although the specific role of lactate in driving adverse clinical outcomes is not fully understood, elevated lactate concentrations are associated with increased morbidity and mortality [15]. Studies have demonstrated that lactate serves as a key indicator for severe sepsis, trauma, and treatment efficacy [16]. A recent study reported that in patients with SALI, a higher lactate-to-albumin ratio was associated with an increased risk of all-cause mortality within 28 days of hospital admission [17]. Wei et al. [18]. identified distinct sepsis subtypes and developed a group-based trajectory model (GBTM) of lactate trajectories for patient risk stratification. However, the association of lactate trajectories with clinical outcomes in patients with SALI remains unclear.

The objective of this study was to assess the clinical prognosis of SALI patients in the ICU by establishing different lactate trajectory subtypes using latent class trajectory modeling (LCTM).

## 2. Materials and Methods

### 2.1. Subjects

The MIMIC-IV dataset includes data from patients hospitalized in the ICU of Beth Israel Deaconess Medical Center between 2008 and 2022 [19]. The data comprise information on vital signs, laboratory test results, patient medications and imaging reports, and admission and discharge information. One of the authors of the present study (C Zhang) completed the web-based National Institutes of Health course “Protecting Human Research Participants” (Record ID: 47400446) and was approved to access the database for research.

The inclusion criteria for patients were those diagnosed with sepsis 3.0, with SOFA scores ≥2, over the age of 18 years, and who had stayed in the ICU for ≥72 h. The exclusion criteria were: patients below the age of 18 years, length of ICU stay < 72 h, a history of chronic liver disease, including cirrhosis, autoimmune hepatitis, or malignant liver tumors, infection with human immunodeficiency virus (HIV), and those who did not undergo daily lactate measurements during the first 96 h following ICU admission.

This study adapted the drug-induced liver injury (DILI) criteria to identify SALI after ICU admission with sepsis [20,21]. The following criteria were used for defining SALI: Alanine aminotransferase (ALT) level at least five times the upper limit of normal (ULN); alkaline phosphatase (ALP) level at least twice the ULN; total bilirubin at least 2.0 mg/dL and ALT at least three times the ULN; total bilirubin at least 2.0 mg/dL and ALP at or above the ULN.

### 2.2. Data Collection and Outcomes

The following basic information of patients with SALI were included: demographic data (sex, age), vital signs (heart rate, mean arterial pressure [MAP], respiratory rate, temperature), laboratory test results (fibrinogen [FIB], prothrombin time [PT], partial thromboplastin time [PTT], international standardized ratio [INR], pH, albumin [ALB], base excess [BE], anion gap [AG], latelets, blood urine nitrogen [BUN], sodium, glucose, creatinine, lactate dehydrogenase [LDH], ALT, aspartate aminotransferase [AST], ALP, bilirubin total [TBIL], white blood cell [WBC], red cell distribution width [RDW], red blood cell [RBC], hemoglobin, and platelet [PLT]), complications (congestive heart failure, diabetes, renal disease, chronic pulmonary disease, metastatic solid tumor), and severity scores (Acute Physiology Score III [APS III] measured on the first day of ICU admission, the highest Sequential Organ Failure Assessment [SOFA] score measured within the first 48 h of ICU admission, Logistic Organ Dysfunction Score [LODS] score max, and Charlson Comorbidity Index [CCI]). All this information was collected within 24 h of ICU admission. The outcome of the study was 30-day survival.

### 2.3. Data Management

The rate of missing data was shown in Supplementary Table S1. The study used a decision tree-based multivariate multiple imputation algorithm to impute missing data. Averaged daily measurements were extracted for assessment of the longitudinal blood lactate trajectories. Patients with no detectable lactate values were excluded from analysis; therefore, no imputation was performed on lactate data. Variables with over 30% missing values were excluded. For the remaining missing data, imputation was performed using the Random Forest algorithm implemented in the “missForest” package in R. LCTM was performed using the “lcmm” R package, and Cox proportional hazards regression models were developed using the “survival” package. XGBoost models were trained using the “xgboost” package, and the SHapley Additive exPlanations (SHAP) algorithm implementation was performed using the “SHAPforxgboost” package. All statistical analyses were performed in R (version 4.5.0).

### 2.4. Statistical Analysis

#### 2.4.1. Latent Class Trajectory Model Development

LCTM was used to examine variations in blood lactate levels in the study participants from day 1 to day 4. LCTM is an unsupervised clustering approach based on longitudinal data that is used to identify clinically distinct subpopulations (latent classes) sharing similar temporal change patterns [22,23]. This method enables the identification of distinct subpopulations, with each characterized by homogeneous trajectory features associated with lactate levels. Model selection was based on a comprehensive evaluation of fit statistics, including the Akaike Information Criterion (AIC), Bayesian Information Criterion (BIC), Sample-adjusted Bayesian Information Criterion (SABIC), and Entropy values. To evaluate the discriminatory capability of the model, the mean posterior class membership probability (MPCMP) was applied as a classification-specific metric, representing the average probability of correct assignment for patients allocated to a given class [24]. The probability that a patient belonged to a specific class was then assessed using this model, ensuring that each trajectory group comprised more than 2% of participants and that the posterior probabilities by class (>0.70).

#### 2.4.2. Statistical Methods

Normally distributed continuous data are presented as mean ± standard deviation (SD). Continuous variables with skewed distributions are reported as median (interquartile range [IQR], Q1–Q3). For multi-group comparisons, the Kruskal–Wallis test was applied for non-parametric data, while analysis of variance (ANOVA) was used for assessing between-group differences. Categorical data are expressed as *n* (%), and were analyzed using Pearson’s chi-square test or Fisher’s exact probability test. Survival was analyzed using Kaplan–Meier curves, with Cox proportional hazards regression models used to examine survival time and associated predictors. Two-sided *p*-values < 0.05 were considered statistically significant.

#### 2.4.3. Analysis of Specific Subclass Interpretability

Two subgroups of SALI patients with moderately elevated lactate levels that showed no significant decline were identified. Both subgroups were associated with higher mortality risk. As the lactate levels fluctuated substantially over 96 h in SALI patients in the ICU, baseline lactate values alone could not effectively distinguish these individuals. A binary XGBoost classification model was therefore developed using baseline clinical data. Hyperparameters were optimized via five-fold cross-validation. Model performance was evaluated using the area under the receiver operating characteristic curve (AUROC), with interpretations generated via SHAP analysis.

## 3. Results

### 3.1. Subjects and Baseline Characteristics

**Figure 1:**
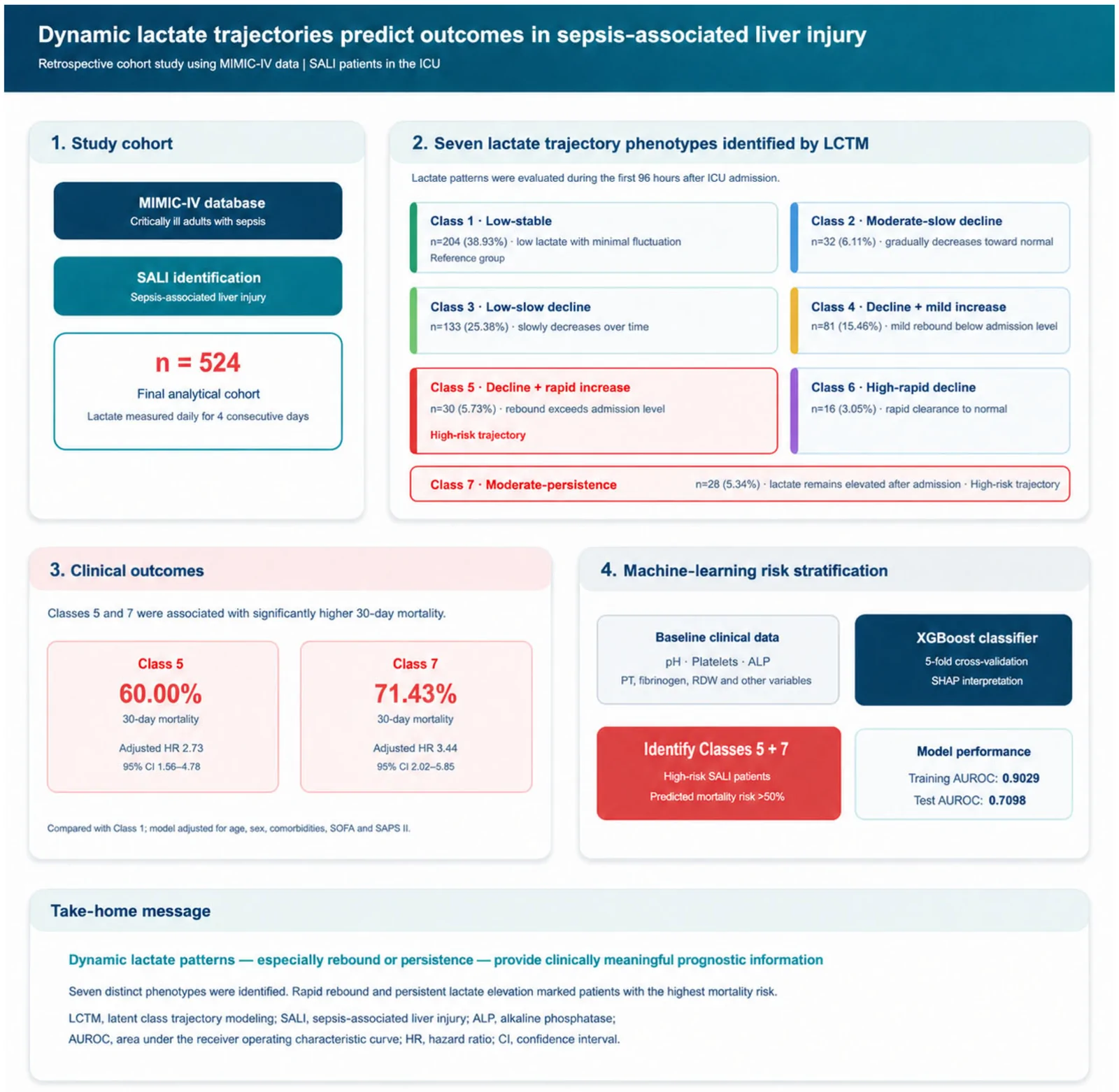
Dynamic lactate patterns predict outcomes in patients with sepsis-associated liver injury.

A total of 3922 patients in the MIMIC-IV dataset were diagnosed with SALI during ICU hospitalization. After application of the exclusion criteria, 524 patients were included in the cohort study (Figure 1). The mean age of the subjects was 62.22 ± 16.77 years, and 61.26% of the patients were male. The overall 30-day mortality rate was 38.74%, with 321 patients surviving and 203 patients dying. The SOFA max score was 10.00 (7.00, 12.00), the length of ICU stay was 9.80 (5.77, 15.95) days, with an in-ICU mortality of 34.16% and an in-hospital mortality of 40.27%. Among these patients, 93.89% received mechanical ventilation (Table 1).

### 3.2. Lactate Trajectory Analysis

**Figure 2:**
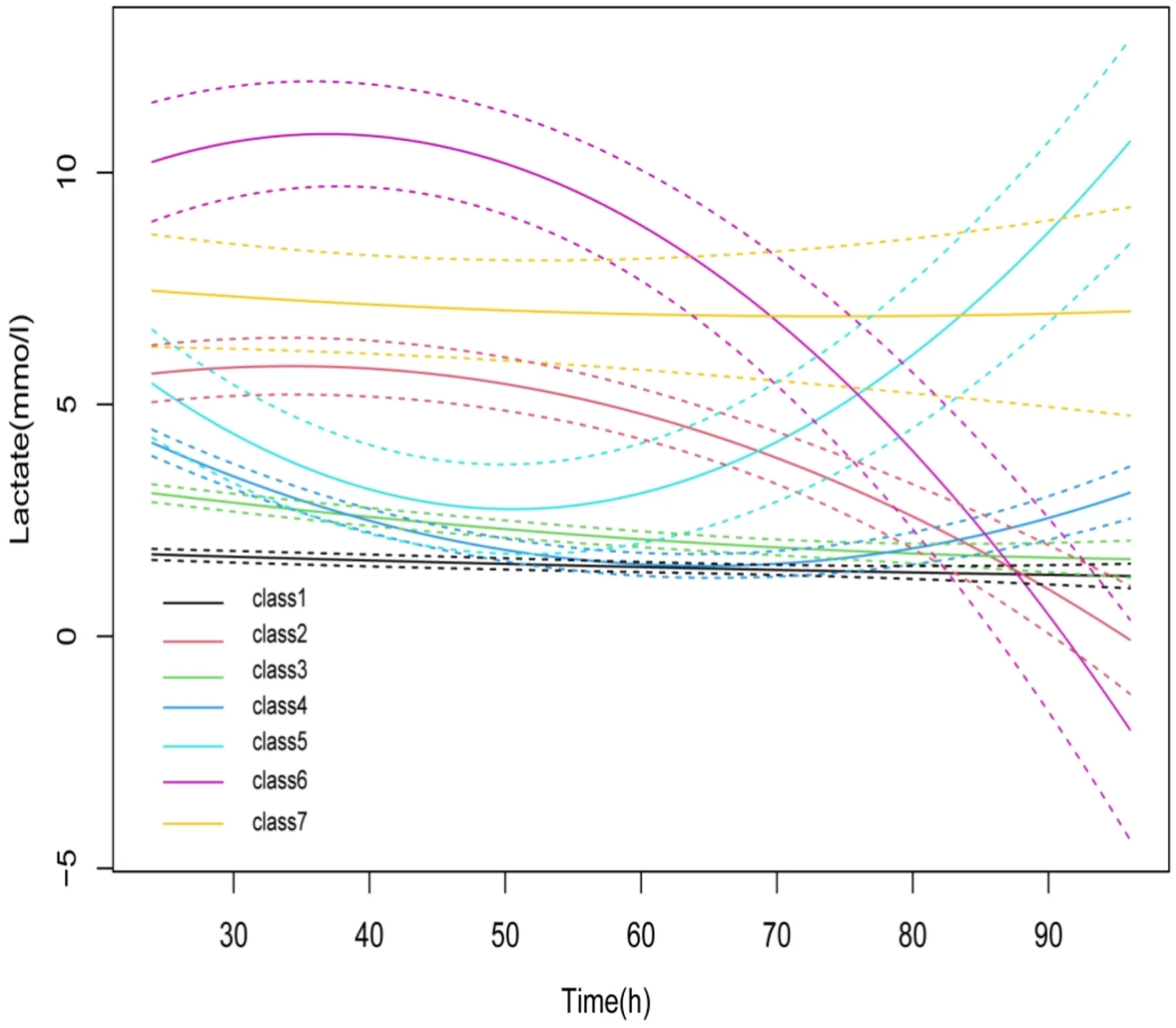
Critically ill patients with SALI classification based on lactate trajectories in MIMIC-IV during the first 96 h of ICU admission. *X*-axis (hours): The difference between the lactate measurement time and the time to ICU admission; *Y*-axis: lactate level.

We fitted latent class trajectory models with 1 to 7 classes and selected the optimal model based on BIC, entropy, average posterior probability, and clinical interpretability (Supplementary Table S2). Although the 6-class model had the lowest BIC (9987.79), the 7-class model showed a higher entropy value (0.8571), and all classes had MPCMP above 0.85, indicating clear and reliable classification (Supplementary Table S3). More importantly, the 7-class model identified a unique trajectory with significant clinical relevance (class 7), which was not found in the 6-class model and holds great value for early intervention and risk stratification. Therefore, considering both statistical indicators and clinical utility, we ultimately selected the 7-class model. The SALI patients in the seven lactate trajectory classes showed significant differences in baseline characteristics and clinical outcomes (Figure 2).

The seven lactate trajectory classes were:

Class 1 (*n* = 204, 38.93%): Low-stable group, characterized by low lactate levels (<2 mmol/L) at ICU admission with minimal subsequent fluctuation;

Class 2 (*n* = 32, 6.11%): Moderate-slow decline group, characterized by moderately elevated lactate levels (≥4 and <8 mmol/LL) that decreased gradually to normal levels.

Class 3 (*n* = 133, 25.38%): Low-slow decline group, characterized by mildly elevated lactate levels (≥2 and <4 mmol/L) that subsequently decreased slowly.

Class 4 (*n* = 81, 15.46%): Moderate-slow decline and mild increase group, characterized by moderately elevated lactate levels (≥4 and <8 mmol/L), followed by a slow decline to within normal limits and a subsequent mild rebound that remained below admission-level hyperlactatemia.

Class 5 (*n* = 30, 5.73%): Moderate-slow decline and rapid increase group, which initially showed moderately elevated lactate levels (≥4 and <8 mmol/L) that declined gradually, followed by a rapid rise that exceeded initial admission levels.

Class 6 (*n* = 16, 3.05%): High-rapid decline group, demonstrating markedly elevated initial lactate levels (≥8 mmol/L), followed by a rapid decline to normal levels.

Class 7 (*n* = 28, 5.34%): Moderate-persistence group, characterized by moderately elevated initial lactate levels (≥4 and <8 mmol/L) that persisted post-admission.

The most prevalent classes were Class 1 (stable low lactate levels), followed by Class 3 (low-level lactate with progressive decline) and Class 4 (moderately elevated lactate with initial decline and mild rebound).

### 3.3. Clinical Characteristics of the Different Subclasses

The clinical characteristics of the different SALI subclasses are shown in Table 1. There were significant differences among subclasses in terms of heart rate, temperature, FIB, PT, PTT, INR, ALB, PH, BE, lactate, AG, glucose, LDH, ALP, ALT, AST, TBIL, WBC, RDW, platelet, RBC, hemoglobin, vasopressin used, hormones used, and presence of metastatic solid tumors. Compared to the overall cohort levels (mean value), Class 1 showed lower levels of PT, INR, lactate, ALT, and AST. Classes 2 and 3 showed higher levels of AST and ALT, while Class 4 had higher PT, INR, lactate, ALT, AST, and WBC values. Class 5 showed the highest lactate, INR, WBC, and PT values, with the lowest pH. Class 6 had the highest TBIL and ALT levels and also exhibited the highest usage of vasopressin and hormones. Class 7 had higher lactate and TBIL levels, but lower levels of ALT and AST.

### 3.4. Analysis of Clinical Outcomes

**Figure 3:**
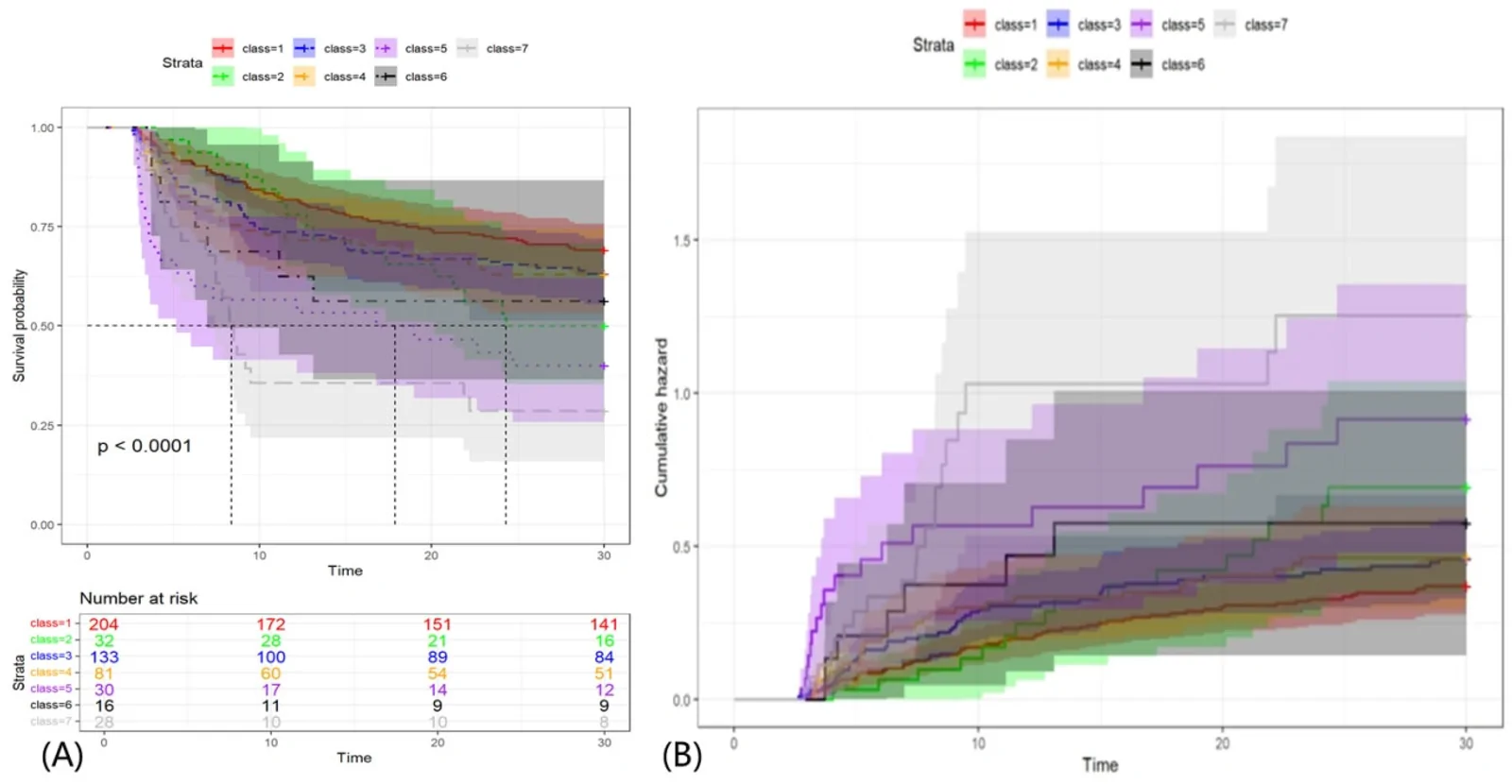
Kaplan–Meier survival curve by the subclasses: (**A**) Kaplan–Meier curves for 30 days. (**B**) Cumulative risk curves for 30 days.

Survival outcomes were compared among the subgroups using Kaplan–Meier curves. As shown in Figure 3A, significant differences in 30-day mortality were observed among the seven classes (log-rank test, *p* < 0.001). The cumulative risk curves indicated a continuously increasing cumulative risk of death over time for patients in Class 7 (Figure 3B), while patients in Class 1 showed the slowest increase in cumulative mortality risk.

### 3.5. Sensitivity Analysis

Four Cox regression models were constructed to verify the associations between lactate trajectories and clinical outcomes, using Class 1 as the reference. In the unadjusted Model 1, Class 5 (hazard ratio [HR] = 2.81, 95% confidence interval [CI]: 1.66–4.74, *p* < 0.001) and Class 7 (HR = 3.57, 95% CI: 2.16–5.92, *p* < 0.001) showed a significantly higher risk of 30-day mortality compared to the other four groups (all *p* < 0.05). In Model 2, adjusted for age and sex, Class 5 (HR = 2.89, 95% CI: 1.70–4.91, *p* < 0.001) and Class 7 (HR = 3.48, 95% CI: 2.10–5.77, *p* < 0.001) maintained a statistically significant elevation in 30-day mortality risk. In Model 3, adjusted for the same parameters as Model 2 with additional adjustments for the comorbidities of chronic pulmonary disease, congestive heart failure, diabetes, and renal disease, Class 5 (HR = 2.89, 95% CI: 1.69–4.96, *p* < 0.001) and Class 7 (HR = 3.61, 95% CI: 2.14–6.06, *p* < 0.001) maintained a statistically significant elevation in 30-day mortality risk. In the fully adjusted Model 4, adjusted for the same parameters as Model 3 and further adjustments for the SOFA and SAPSII scores, Class 5 (HR = 2.73, 95% CI: 1.56–4.78, *p* < 0.001) and Class 7 (HR = 3.44, 95% CI: 2.02–5.85, *p* < 0.001) continued to show significantly raised 30-day mortality risks. Although AKI stage was recorded, it was not included in the primary Cox models because of its limited variability across trajectory classes and potential overlap with SOFA-based organ dysfunction assessment. A sensitivity analysis additionally adjusting for AKI stage was performed to evaluate the robustness of the findings. Classes 2, 3, 4, and 6 exhibited hazard ratios greater than 1 (HR > 1), indicating a potentially increased mortality risk relative to the reference Class 1; however, these associations did not achieve statistical significance in any of the models (all *p* > 0.05). The complete results are presented in Table 2.

### 3.6. Model Performance and Explanation for Classes 5 and 7

Patients in Classes 5 and 7 were defined as Group 1 with a >50% 30-day mortality risk, while those in Classes 1–4 and 6 were classified as Group 0. The results of the initial univariate analysis indicated statistically significant differences between the two groups (Supplementary Table S4). Subsequent collinearity diagnostics revealed severe multicollinearity between hemoglobin and RBC, with variance inflation factors (VIFs) >8.9, and between pH and BE (VIF > 2.8). After excluding RBC and BE, the residual collinearity patterns were confirmed using variance inflation analysis (Supplementary Figure S1) and correlation matrices (Supplementary Figure S2).

**Figure 4:**
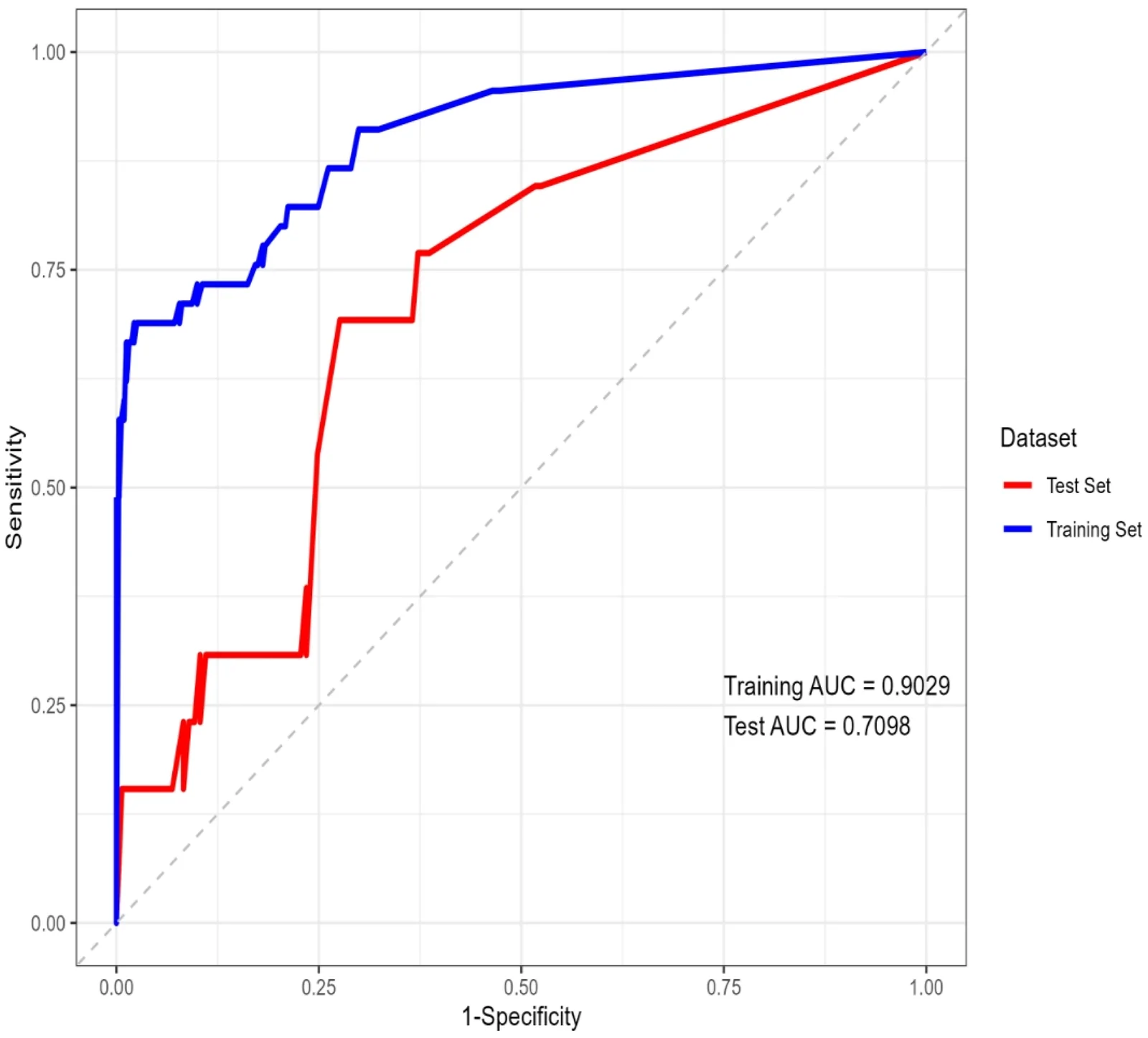
ROC curves for Training and Test Sets.

**Figure 5:**
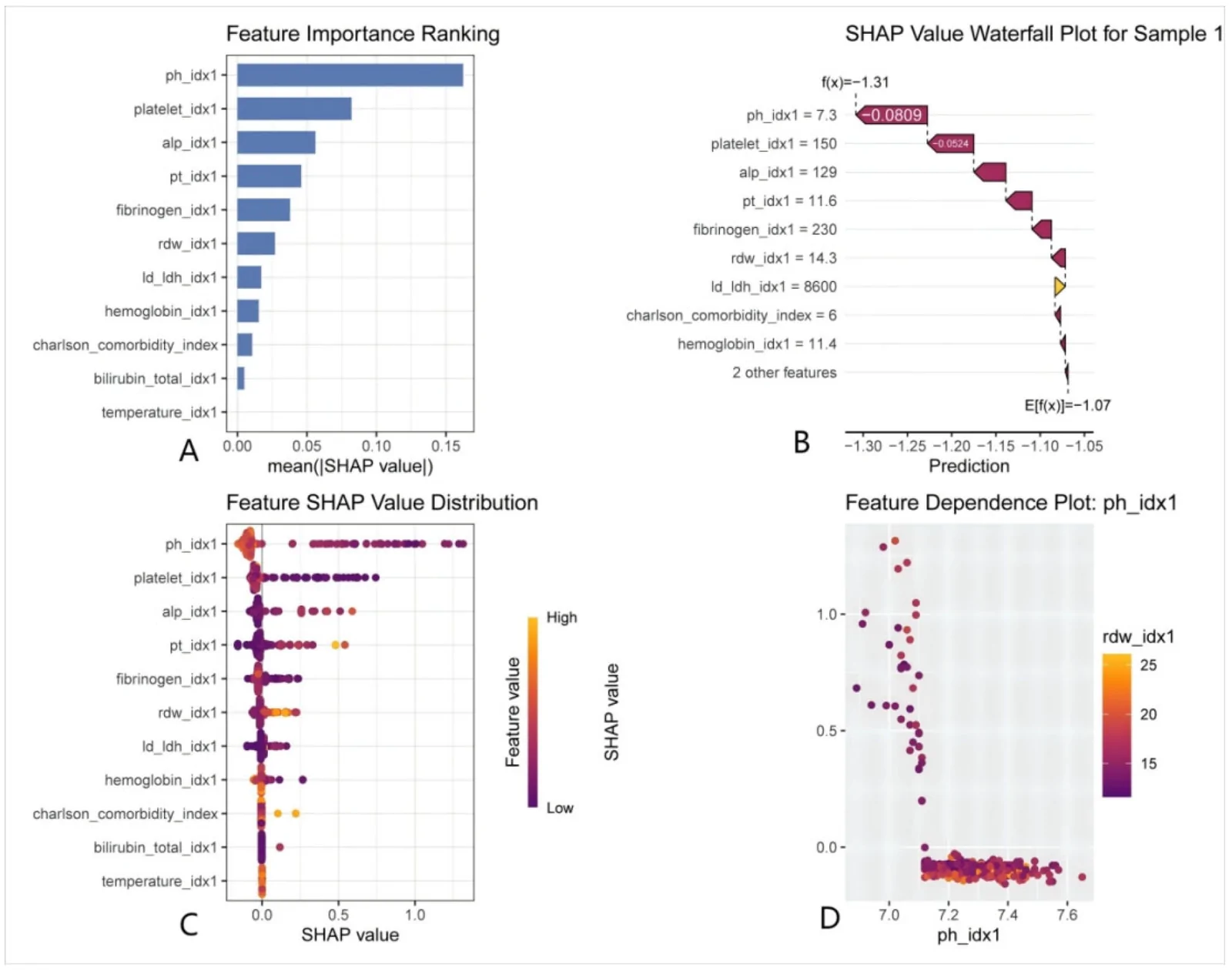
Visually interpret machine learning models using SHAP: (**A**) SHAP feature importance plot. (**B**) SHAP value waterfall plot. (**C**) SHAP summary plot. (**D**) Feature dependence plot.

The XGBoost classifier was trained on a 70% training subset of the cohort to identify patients labeled as “Class 5 and 7” (high-risk), with hyperparameter optimization performed via five-fold cross-validation. On the held-out test set (30% of the cohort), the model achieved a moderate discriminative performance, with an AUROC of 0.7098, compared to a training AUROC of 0.9029, indicating limited generalizability likely due to overfitting (Figure 4). SHAP analysis was used for exploratory purposes to identify potential predictors: the feature importance plot (Figure 5A) and summary plot (Figure 5C) demonstrated the different influences on the model. In addition, force plots were used to visualize the predictions of the individual models in terms of feature contributions (Figure 5B). To validate the observed interaction between PH and RDW, a supplementary interaction SHAP plot was generated (Figure 5D).

## 4. Discussion

This study analyzed data from the MIMIC-IV dataset using LCTM. This is the first demonstration that dynamic lactate measurements were significantly predictive of mortality in critically ill patients with SALI. Seven distinct lactate trajectory phenotypes were identified. Among these, Class 5 (moderate-slow decline and rapid increase) and Class 7 (moderate-persistence) emerged as high-risk signatures, demonstrating 2.7–3.6 times higher 30-day mortality compared to Class 1 (low-stable group) after comprehensive adjustment (HR: 3.44, 95% CI: 2.02–5.85; *p* < 0.001). This association was maintained in various sequential multivariate models with adjustments for demographic factors, chronic comorbidities (pulmonary, cardiac, and renal disease and diabetes), and illness severity scores (SOFA/SAPSII). Strikingly, while Class 6 showed the highest initial lactate levels among the different subgroups, it also demonstrated rapid clearance, resulting in significantly reduced 30-day mortality (HR 1.69; 95% CI 0.77–3.69, *p* = 0.188). The proportion of hormonal agents to vasopressin required in Class 6 was notably higher compared to that in the other Classes. These findings suggest that: (1) the measurement of lactate trajectories is superior to that of isolated values in evaluating prognosis; (2) early correction of hyperlactatemia in SALI is independently predictive of improved survival. However, it is important to note that these associations are derived from observational data and do not imply causality. The higher proportion of vasopressin use observed in Class 6, which exhibited rapid lactate clearance and favorable outcomes, should be considered a hypothesis-generating finding. Whether vasopressin or other trajectory-guided interventions directly improve outcomes in SALI patients requires validation in prospective studies or randomized controlled trials. The identification of lactate trajectory subtypes not only provides prognostic insights but also holds significant clinical utility as a risk stratification tool. For patients exhibiting persistently elevated lactate levels or a sudden surge following a slow decline, intensified monitoring, personalized hemodynamic support, or early therapeutic modifications may be warranted. Furthermore, the results demonstrate that trajectory modeling is a practical strategy for critical care decision-making.

There has been recent interest in the identification of biomarkers for the early detection of sepsis patients with high mortality risk [25]. Notably, the use of lactate dynamics has already been shown to be linked with prognosis in critically ill patients, a relationship validated in clinical studies of cirrhosis-related critical illness [26] and acute liver failure [27]. The liver, the primary metabolic organ for lactate clearance, typically converts 70% of systemic lactate into glucose via gluconeogenesis [28]. However, SALI disrupts this metabolic pathway, precipitating pathological hyperlactatemia. This hyperlactatemia can originate from (1) hypoperfusion-induced anaerobic glycolysis, (2) catecholamine-driven aerobic glycolysis, (3) impaired hepatic lactate clearance, and (4) mitochondrial pyruvate dehydrogenase (PDH) suppression [29]. In this study, Class 7 showed enzyme-bilirubin dissociation, indicating that elevated lactate levels in this phenotype resulted primarily from acute injury-induced disruption of hepatic metabolism.

The study found that dynamic changes in blood lactate concentrations during the early phase of sepsis following admission may be a readily accessible biomarker for elevated mortality risk. Intriguingly, this hypothesis is substantiated by the observation that early goal-directed resuscitation in patients with septic shock is effective in improving lactate clearance [30]. Studies have shown that the lactate-to-albumin ratio (LAR) represents an independent risk factor for 28-day all-cause mortality in SALI patients, with the risk of mortality increasing progressively with higher LAR values. However, this previous research focused solely on the correlation between the initial LAR recorded on admission and 28-day mortality, and did not analyze dynamic relationships. The present study addressed this critical knowledge gap through longitudinal analysis [17]. A multicenter retrospective observational analysis of 10,724 patients with sepsis reported that the mortality rate for those with severe hyperlactatemia that did not reduce within the first 24 h was 92% [31]. Another retrospective observational study analyzed 5041 patients during the first 24 h of ICU admission. Both the time-weighted average lactate (LACTW24) and the change in lactate (LACΔ24) levels over the first 24 h were found to be independently predictive of hospital mortality, with both showing linear relationships [32]. While these two studies investigated the correlation between 24 h changes in lactate levels and prognosis in critically ill patients, the present analysis extended the observation window to 96 h, thereby complementing and substantiating the existing evidence. Dynamic lactate trajectory modeling is increasingly employed in clinical research settings. A study of MIMIC-IV data employing group-based trajectory modeling (GBTM) revealed that stroke patients with slow-clearance lactate trajectories had a significantly higher likelihood of mortality compared to those with rapid-clearance patterns. An additional three independent cohort studies indicated a strong association between rapid lactate clearance and favorable outcomes in hyperlactatemia-associated patients with acute kidney injury (AKI). Persistently elevated lactate levels were found to significantly increase both mortality risk and the probability of AKI progression [33].

Although the clinical relationship between blood lactate concentration and SALI has been extensively studied, the underlying mechanisms remain unclear. Li et al. found that accumulation of lactate within the hepatic microenvironment was induced by increased expression of caspase-11, leading to cleavage of gasdermin D and increased macrophage pyroptosis, worsening liver damage [34]. There is accumulating evidence that macrophage pyroptosis is mechanistically implicated in the pathophysiology of sepsis [35,36]. Mechanistic investigations have also shown that lactylation participates in sepsis pathogenesis [37] and is closely related to liver damage, demonstrating a strong positive correlation with circulating lactate concentrations [38,39]. Furthermore, the present study found that patients in Class 5 exhibited significant increases in white blood cells, INR, PT, and lactate levels (all *p* < 0.01), together with the lowest MAP, indicating the coexistence of severe systemic inflammation and coagulopathy. Previous studies have revealed that during systemic inflammatory responses, endotoxins such as lipopolysaccharide (LPS) enhance the transcriptional activity of nuclear factor-κB (NF-κB) through activation of the Toll-like receptor 4 (TLR4) signaling pathway [40]. This process induces the expression of Nod-like receptor pyrin domain containing 3 (NLRP3) and pro-interleukin-1β (pro-IL-1β), triggering assembly of the inflammasome. The multiprotein NLRP3 inflammasome complex then activates caspase-1 to induce IL-1β maturation and release, thereby playing a central role in the regulation of innate immune responses in the liver and the pathogenesis of SALI [41]. Notably, persistent LPS stimulation in sepsis establishes a positive feedback loop that amplifies proinflammatory cytokine cascades (including IL-1β, IL-6, and TNF-α) via the NF-κB pathway, ultimately exacerbating liver damage [42]. The increase in lactate levels is associated with mitochondrial damage [43,44], exacerbation of inflammation, and reperfusion oxidant injury, necessitating early intervention to target the source of infection.

Nevertheless, our results mainly apply to critically ill patients who remained under ICU observation and had sufficient lactate measurements to characterize temporal trajectories. Future prospective studies with predefined lactate monitoring protocols or statistical approaches specifically designed to handle informative missingness are warranted to further validate these findings.

In addition, there are several limitations to this study. First, the retrospective design and focus on the acute phase (96 h post-admission) precluded analysis of lactate fluctuations during later stages (e.g., secondary infections, terminal organ failure). Future studies should extend the monitoring window. Second, the use of observational data extracted solely from the MIMIC-IV dataset resulted in a moderate sample size from a single center that was restricted to ICU patients in the USA, potentially limiting the generalizability of the findings to other populations. Future multi-center studies with larger cohorts incorporating international databases or real-world hospital data are needed. We emphasize that external validation in independent multicenter ICU cohorts is required before considering clinical application. Third, common clinical interventions, including the use of vasopressor agents [45], metformin [46], and continuous renal replacement therapy [47], may have influenced the lactate levels. The lack of precise information represents an uncontrolled confounder, necessitating verification using prospective multi-center designs. Fourth, the use of modified DILI criteria may have influenced case identification; we acknowledge that there is no universally accepted operational definition of SALI and that alternative definitions based on bilirubin, aminotransferases, and/or coagulation abnormalities have also been used. Future work should compare multiple definitions. Nevertheless, the study successfully identified reliable subphenotypes of SALI with distinct clinical manifestations and significantly different effects on prognosis. These results not only provide critical insights into SALI progression but also support future ICU-based studies on early subphenotype prediction and the exploration of differential treatment responses.

## 5. Conclusions

In this study, latent class trajectory modeling (LCTM) identified distinct SALI subtypes by dynamic lactate profiling. Classes 5 and 7 were found to be high-risk phenotypes, establishing an actionable framework for stratified risk management in clinical practice. Early targeted intervention based on these predictive trajectories may reduce patient mortality and improve outcomes. These findings provide deeper insights into the factors underlying SALI progression. Future implementation of lactate trajectory phenotyping could enhance prognostic precision in critical care settings. The XGBoost model, however, should be considered an exploratory tool rather than a validated clinical predictor, and its findings require external validation before clinical translation.

## Figures and Tables

**Table 1:** Clinical characteristics between subclasses.

Variables	Total (*n* = 524)	1 (*n* = 204)	2 (*n* = 32)	3 (*n* = 133)	4 (*n* = 81)	5 (*n* = 30)	6 (*n* = 16)	7 (*n* = 28)	*p*
Demographic data									
Gender(%)									0.76
Female	203 (38.74)	80 (39.22)	12 (37.50)	50 (37.59)	28 (34.57)	15 (50.00)	5 (31.25)	13 (46.43)	
Male	321 (61.26)	124 (60.78)	20 (62.50)	83 (62.41)	53 (65.43)	15 (50.00)	11 (68.75)	15 (53.57)	
Age	62.22 ± 16.77	62.00 ± 16.76	66.43 ± 14.11	60.97 ± 17.35	60.87 ± 17.83	63.25 ± 16.84	67.54 ± 11.04	64.66 ± 16.27	0.47
Vital signs									
Heart Rate	98.50 (82.00, 113.00)	96.00 (79.75, 109.00)	107.00 (92.75, 122.75)	100.00 (82.00, 113.00)	103.00 (83.00, 117.00)	89.50 (78.50, 112.00)	94.00 (86.25, 109.25)	103.00 (91.50, 112.00)	0.02
MAP (mmHg)	78.00 (69.00, 91.00)	78.00 (70.00, 91.00)	80.50 (68.25, 89.00)	78.00 (69.00, 92.00)	76.00 (64.00, 100.00)	73.50 (66.00, 85.50)	83.25 (74.50, 87.50)	81.00 (71.00, 102.00)	0.80
Resp Rate	21.00 (17.00, 26.00)	19.00 (16.00, 25.25)	20.50 (17.75, 26.00)	23.00 (18.00, 27.00)	22.00 (16.00, 27.00)	23.00 (19.00, 27.75)	23.50 (17.25, 25.50)	20.50 (19.00, 25.50)	0.11
Temperature	36.50 (36.06, 37.00)	36.56 (36.16, 37.00)	36.67 (36.39, 37.28)	36.56 (36.06, 37.06)	36.44 (35.61, 36.89)	36.25 (34.58, 36.53)	36.80 (36.05, 37.35)	36.55 (36.19, 36.90)	<0.01
Laboratory ^a^									
Fibrinogen	307.68 (187.75, 418.22)	351.59 (250.94, 453.31)	304.00 (201.75, 429.75)	296.00 (185.00, 406.33)	248.00 (174.00, 336.00)	239.00 (156.50, 345.37)	212.00 (157.25, 352.25)	209.50 (152.00, 245.72)	<0.01
PT	17.40 (14.30, 22.22)	15.50 (13.57, 19.70)	17.75 (14.52, 22.15)	17.40 (14.20, 22.20)	18.80 (15.80, 25.00)	20.80 (16.72, 44.10)	18.10 (16.93, 21.68)	20.50 (16.35, 28.72)	<0.01
PTT	35.50 (29.70, 46.92)	32.85 (28.28, 41.50)	37.60 (29.30, 50.60)	37.00 (30.40, 46.50)	40.10 (31.60, 55.00)	38.65 (31.00, 54.65)	33.55 (30.50, 50.47)	35.20 (30.60, 47.85)	<0.01
INR	1.60 (1.30, 2.02)	1.40 (1.20, 1.80)	1.65 (1.30, 2.02)	1.60 (1.30, 2.00)	1.70 (1.40, 2.30)	1.90 (1.52, 3.68)	1.70 (1.58, 2.02)	1.85 (1.50, 2.60)	<0.01
Albumin (g/dL)	2.70 (2.30, 3.10)	2.70 (2.30, 3.10)	2.45 (1.90, 3.10)	2.80 (2.40, 3.13)	2.80 (2.32, 3.30)	2.90 (2.50, 3.25)	2.20 (2.00, 2.52)	2.75 (2.20, 3.32)	0.01
PH	7.29 (7.20, 7.37)	7.32 (7.25, 7.39)	7.30 (7.20, 7.40)	7.29 (7.21, 7.34)	7.22 (7.12, 7.29)	7.21 (7.06, 7.31)	7.31 (7.23, 7.39)	7.30 (7.13, 7.36)	<0.01
Base Excess (mmol/L)	−6.00 (−10.00, −2.00)	−3.00 (−7.00, 0.00)	−6.00 (−9.25, −2.00)	−6.00 (−10.00, −3.00)	−10.00 (−14.00, −6.00)	−14.50 (−19.00, −7.75)	−6.50 (−10.25, −2.75)	−8.00 (−15.25, −3.00)	<0.01
Lactate (mmol/L)	3.40 (1.90, 6.12)	1.80 (1.50, 2.40)	3.50 (2.10, 5.10)	4.00 (3.40, 4.50)	7.80 (7.20, 9.40)	12.80 (10.18, 14.82)	6.60 (3.25, 7.55)	8.35 (5.60, 10.20)	<0.001
Aniongap	19.00 (15.00, 23.00)	16.00 (13.00, 18.25)	18.00 (14.75, 22.25)	19.00 (17.00, 22.00)	22.00 (18.00, 26.00)	31.00 (23.50, 34.00)	20.50 (18.00, 26.50)	24.00 (20.50, 29.25)	<0.01
BUN(mg/dL)	31.00 (19.00, 49.00)	31.00 (20.00, 52.00)	32.50 (21.75, 46.00)	29.00 (19.00, 55.00)	30.00 (19.00, 43.00)	34.00 (21.50, 48.25)	27.00 (15.75, 40.00)	36.50 (21.00, 50.00)	0.73
Sodium (mmol/L)	138.00 (134.00, 142.00)	137.00 (134.00, 141.00)	139.00 (133.00, 142.00)	138.00 (134.00, 143.00)	139.00 (136.00, 142.00)	140.00 (137.25, 143.50)	138.50 (135.50, 141.00)	139.00 (135.00, 140.25)	0.21
Glucose (mg/dL)	151.50 (114.75, 217.00)	143.50 (111.00, 187.25)	182.00 (133.75, 215.50)	168.00 (126.00, 263.00)	196.00 (128.00, 284.00)	174.00 (107.00, 247.00)	124.00 (88.25, 155.25)	129.00 (101.25, 190.00)	<0.01
Creatinine (mg/dL)	1.60 (1.10, 2.80)	1.50 (1.00, 2.68)	1.50 (1.20, 2.47)	1.60 (1.20, 2.80)	1.60 (1.20, 2.40)	2.20 (1.60, 3.68)	1.45 (1.00, 1.97)	1.70 (1.00, 2.92)	0.09
Ld	806.00 (451.00, 1958.75)	572.50 (331.75, 973.00)	1047.50 (487.25, 1920.50)	943.00 (523.00, 2266.00)	1610.00 (730.00, 3775.00)	1298.00 (613.75, 2308.00)	892.50 (600.00, 4473.75)	1608.53 (791.50, 3598.75)	<0.01
ALT(U/L)	337.00 (132.00, 856.00)	230.00 (43.50, 443.50)	407.00 (61.50, 615.00)	489.00 (243.00, 1223.00)	570.00 (256.00, 1613.00)	596.00 (269.50, 1197.50)	598.50 (195.50, 1557.75)	215.50 (53.00, 659.00)	<0.01
ALP(U/L)	116.00 (70.00, 226.50)	139.50 (73.75, 274.00)	112.50 (60.00, 259.00)	101.00 (70.00, 160.00)	88.00 (52.00, 151.00)	125.00 (78.25, 216.75)	99.50 (68.75, 266.75)	244.50 (117.75, 345.00)	<0.01
AST(U/L)	483.00 (174.25, 1476.50)	255.00 (80.75, 685.00)	664.50 (87.25, 1383.50)	803.00 (252.00, 1771.00)	940.00 (378.00, 2834.00)	973.00 (414.25, 2109.50)	996.00 (326.75, 2351.75)	380.50 (113.50, 1385.00)	<0.01
Bilirubin Total (mg/dL)	1.30 (0.70, 2.92)	1.20 (0.60, 2.70)	1.20 (0.78, 2.92)	1.30 (0.80, 2.50)	1.00 (0.60, 2.40)	1.35 (0.83, 2.68)	3.10 (1.65, 4.03)	2.60 (1.00, 7.65)	<0.01
White Blood Cells(×10^9^/L)	14.30 (9.88, 20.22)	13.80 (9.80, 19.52)	14.25 (10.10, 18.20)	14.20 (9.90, 20.10)	15.80 (11.10, 20.40)	20.95 (12.90, 26.40)	10.40 (6.10, 12.85)	10.65 (6.20, 16.65)	<0.01
RDW	15.10 (13.90, 16.90)	15.20 (14.10, 16.90)	15.45 (13.95, 17.40)	14.80 (13.80, 16.50)	14.40 (13.70, 15.50)	16.40 (14.88, 18.95)	16.30 (15.05, 17.35)	15.85 (14.73, 17.50)	<0.01
Red Blood Cells (×10^12^/L)	3.59 (3.00, 4.22)	3.54 (2.96, 4.14)	3.79 (2.96, 4.17)	3.79 (3.22, 4.43)	3.70 (3.07, 4.43)	3.29 (2.79, 3.81)	3.20 (3.02, 3.72)	3.26 (2.69, 4.00)	0.02
Platelet (×1000/mm^3^)	167.00 (110.00, 249.00)	185.50 (124.50, 263.50)	199.50 (133.75, 267.00)	164.00 (107.00, 228.00)	157.00 (114.00, 243.00)	202.00 (130.25, 269.50)	80.00 (40.25, 119.75)	79.50 (37.00, 157.25)	<0.01
Hemoglobin (g/dL)	10.70 (8.90, 12.70)	10.40 (9.00, 12.53)	11.30 (8.40, 12.50)	11.30 (9.70, 13.50)	11.00 (9.60, 13.50)	9.45 (7.90, 11.73)	10.20 (9.35, 11.70)	10.25 (8.38, 11.27)	<0.01
Comorbidities									
Congestive Heart Failure, *n* (%)									0.16
No	309 (58.97)	122 (59.80)	17 (53.12)	72 (54.14)	50 (61.73)	15 (50.00)	10 (62.50)	23 (82.14)	
Yes	215 (41.03)	82 (40.20)	15 (46.88)	61 (45.86)	31 (38.27)	15 (50.00)	6 (37.50)	5 (17.86)	
Diabetes, *n* (%)									0.91
No	351 (66.98)	135 (66.18)	21 (65.62)	88 (66.17)	55 (67.90)	19 (63.33)	13 (81.25)	20 (71.43)	
Yes	173 (33.02)	69 (33.82)	11 (34.38)	45 (33.83)	26 (32.10)	11 (36.67)	3 (18.75)	8 (28.57)	
Renal Disease, *n* (%)									0.8
No	395 (75.38)	154 (75.49)	22 (68.75)	103 (77.44)	64 (79.01)	20 (66.67)	12 (75.00)	20 (71.43)	
Yes	129 (24.62)	50 (24.51)	10 (31.25)	30 (22.56)	17 (20.99)	10 (33.33)	4 (25.00)	8 (28.57)	
Chronic Pulmonary Disease, *n* (%)									0.14
No	376 (71.76)	133 (65.20)	23 (71.88)	100 (75.19)	61 (75.31)	26 (86.67)	13 (81.25)	20 (71.43)	
Yes	148 (28.24)	71 (34.80)	9 (28.12)	33 (24.81)	20 (24.69)	4 (13.33)	3 (18.75)	8 (28.57)	
Metastatic Solid Tumor, *n* (%)									<0.01
No	488 (93.13)	192 (94.12)	30 (93.75)	126 (94.74)	79 (97.53)	27 (90.00)	13 (81.25)	21 (75.00)	
Yes	36 (6.87)	12 (5.88)	2 (6.25)	7 (5.26)	2 (2.47)	3 (10.00)	3 (18.75)	7 (25.00)	
AKI Stage	3.00 (2.00, 3.00)	3.00 (2.00, 3.00)	3.00 (3.00, 3.00)	3.00 (2.00, 3.00)	3.00 (3.00, 3.00)	3.00 (3.00, 3.00)	3.00 (3.00, 3.00)	3.00 (3.00, 3.00)	<0.01
Ventilator Flag, *n* (%)									<0.01
No	32 (6.11)	5 (2.45)	1 (3.12)	12 (9.02)	8 (9.88)	1 (3.33)	0 (0.00)	5 (17.86)	
Yes	492 (93.89)	199 (97.55)	31 (96.88)	121 (90.98)	73 (90.12)	29 (96.67)	16 (100.00)	23 (82.14)	
Treatment									
Vasopressin ^b^ Used (%)									<0.01
No	285 (54.39)	127 (62.25)	10 (31.25)	74 (55.64)	45 (55.56)	16 (53.33)	3 (18.75)	10 (35.71)	
Yes	239 (45.61)	77 (37.75)	22 (68.75)	59 (44.36)	36 (44.44)	14 (46.67)	13 (81.25)	18 (64.29)	
Hormone ^c^ Used (%)									<0.01
No	361 (68.89)	149 (73.04)	21 (65.62)	100 (75.19)	57 (70.37)	16 (53.33)	4 (25.00)	14 (50.00)	
Yes	163 (31.11)	55 (26.96)	11 (34.38)	33 (24.81)	24 (29.63)	14 (46.67)	12 (75.00)	14 (50.00)	
ICU disease severity scores									
APS III Max	72.00 (56.00, 91.00)	62.00 (50.75, 84.00)	83.50 (66.75, 103.00)	72.00 (60.00, 88.00)	79.00 (61.00, 92.00)	88.00 (75.25, 108.75)	89.50 (68.50, 95.25)	76.00 (54.00, 97.00)	<0.01
LODS Max	9.00 (7.00, 10.00)	8.00 (6.00, 10.00)	10.00 (8.00, 11.00)	9.00 (7.00, 10.00)	9.00 (7.00, 11.00)	10.00 (8.25, 12.75)	9.50 (8.75, 11.00)	9.00 (6.00, 11.00)	<0.01
SOFA Max	10.00 (7.00, 12.00)	9.00 (6.75, 11.00)	11.00 (8.75, 13.25)	10.00 (8.00, 12.00)	11.00 (9.00, 13.00)	10.50 (8.25, 13.75)	13.00 (11.25, 15.00)	9.50 (7.00, 14.25)	<0.01
SAPS II Max	53.00 (42.00, 64.00)	48.00 (38.75, 60.00)	60.50 (48.75, 65.75)	54.00 (43.00, 64.00)	54.00 (44.00, 66.00)	60.00 (49.50, 71.75)	61.00 (52.25, 69.25)	56.50 (47.75, 69.50)	<0.01
Charlson Comorbidity	5.00 (3.00, 8.00)	5.00 (3.00, 8.00)	6.00 (3.00, 8.00)	5.00 (2.00, 7.00)	5.00 (3.00, 6.00)	6.00 (3.00, 8.75)	6.00 (4.00, 8.00)	6.50 (4.00, 9.25)	0.08
Hosp Outcome 30 d (%)									<0.01
Survival	321 (61.26)	141 (69.12)	16 (50.00)	84 (63.16)	51 (62.96)	12 (40.00)	9 (56.25)	8 (28.57)	
Non-survival	203 (38.74)	63 (30.88)	16 (50.00)	49 (36.84)	30 (37.04)	18 (60.00)	7 (43.75)	20 (71.43)	
LOS Icu	9.80 (5.77, 15.95)	9.99 (6.07, 15.58)	11.00 (6.63, 19.77)	10.76 (5.91, 16.02)	9.54 (6.02, 15.99)	6.75 (3.81, 9.92)	11.13 (4.68, 22.29)	6.33 (4.36, 13.04)	0.03
Hosp Time 30 d	30.00 (10.42, 30.00)	30.00 (19.05, 30.00)	27.16 (13.03, 30.00)	30.00 (10.00, 30.00)	30.00 (9.05, 30.00)	17.87 (3.59, 30.00)	30.00 (6.79, 30.00)	8.37 (5.29, 30.00)	<0.01

^a^ Laboratory values were selected based on the most abnormal results within 24 h of ICU admission. ^b^ Vasopressors included norepinephrine, dopamine, epinephrine, phenylephrine, and vasopressin. ^c^ Hormones included the sum of hydrocortisone, prednisolone, and dexamethasone. MAP, mean arterial pressure; PT, prothrombin time; INR, international normalisation ratio; PTT, partial thromboplastin time; BUN, blood urea nitrogen; LDH, lactate dehydrogenase; ALT, alanine aminotransferase; AST, aspartate aminotransferase; ALP, Alkaline Phosphatase; RDW, red blood cell distribution width; AKI, Acute Kidney Injury; SOFA, sequential organ failure assessment; SAPS II/SAPS III, Simplified Acute Physiology Score II (III); LODS, Logistic Organ Dysfunction System; LOS ICU, Length of Stay in ICU.

**Table 2:** Complete results of Cox regression in subclasses.

Variables	Model 1 [HR (95% CI), *p*-Value]	Model 2 [HR (95% CI), *p*-Value]	Model 3 [HR (95% CI), *p*-Value]	Model 4 [HR (95% CI), *p*-Value]
30-day survival				
Class 1	Reference	Reference	Reference	Reference
Class 2	1.67 (0.96, 2.89), 0.067	1.55 (0.90, 2.69), 0.117	1.56 (0.90, 2.70), 0.152	1.50 (0.86, 2.62), 0.152
Class 3	1.28 (0.88, 1.86), 0.198	1.29 (0.89, 1.87), 0.186	1.26 (0.86, 1.83), 0.282	1.23 (0.84, 1.80), 0.282
Class 4	1.30 (0.85, 2.02), 0.226	1.31 (0.85, 2.02), 0.225	1.33 (0.86, 2.06), 0.282	1.28 (0.82, 1.99), 0.282
Class 5	2.81 (1.66, 4.74), <0.001	2.89 (1.70, 4.91), <0.001	2.89 (1.69, 4.96), <0.001	2.73 (1.56, 4.78), <0.001
Class 6	1.69 (0.77, 3.69), 0.188	1.57 (0.72, 3.43), 0.259	1.52 (0.69, 3.36), 0.389	1.42 (0.64, 3.18), 0.389
Class 7	3.57 (2.16, 5.92), <0.001	3.48 (2.10, 5.77), <0.001	3.61 (2.14, 6.06), <0.001	3.44 (2.02, 5.85), <0.001

Model 1 was a non-adjusted model. Model 2 was adjusted for age and gender. Model 3 was adjusted for the same parameters as Model 2 with additional adjustments for comorbidities of chronic pulmonary disease, congestive heart failure, diabetes, and renal disease. Model 4 was adjusted for the same parameters as Model 3 with additional adjustments for SOFA score and SAPSII score. HR, hazard ratio; 95% CI, 95% confidence interval. The bold values indicate statistically significant values (*p* < 0.05).

## Data Availability

The dataset used in this study from MIMIC-IV is available at https://mimic.physionet.org/.

## Supplementary Materials

The following supporting information can be downloaded at https://www.mdpi.com/article/10.3390/jcm15176837/s1. Figure S1. the Variable Inflation Factor (VIF) analysis results. Figure S2. Correlation cefficient matrix. Table S1. Missing data summary. Table S2. Fit statistics of LCTMs from 1 to 7 subclasses. Table S3. Mean Posterior Probability of Membership in Class in MIMIC-IV. Table S4. Clinical characteristics between Group 0 and Group 1.

## Abbreviations

SALI	Sepsis-associated liver injury
ICU	intensive care unit
GBTM	Group-Based Trajectory Model
LCTM	Latent Class Trajectory Modeling
HIV	human immunodeficiency virus
DILI	drug-induced liver injury
ALT	Alanine aminotransferase
ULN	upper limit of normal
ALP	Alkaline phosphatase
MAP	mean arterial pressure
FIB	fibrinogen
PT	prothrombin time
PTT	partial thromboplastin time
INR	international standardized ratio
ALB	albumin
BE	base excess
BUN	blood urine nitrogen
LDH	lactate dehydrogenase
AST	aspartate aminotransferase
TBIL	bilirubin total
WBC	white blood cell
RDW	red cell distribution width
RBC	red blood cell
APS III	Acute Physiology Score III
SOFA	Sequential Organ Failure Assessment
SHAP	SHapley Additive exPlanations
BIC	Bayesian Information Criterion
MPCMP	mean posterior class membership probability
SD	standard deviation
IQR	interquartile range
ANOVA	analysis of variance
AUROC	Area Under the ROC Curve
HR	hazard ratio
CI	confidence interval
VIF	Variance Inflation Factors
PDH	pyruvate dehydrogenase
LAR	lactate-to-albumin ratio
AKI	acute kidney injury
NF-κB	nuclear factor-κB
TLR4	Toll-like receptor 4
NLRP3	Nod-like receptor pyrin domain containing 3
