# Supplementary material for: Association Between Lactate Trajectories and Clinical Outcomes in Critically Ill Patients with Sepsis-Associated Liver Injury
Source: J Clin Med. 2026 Sep 3;15(17):6837. doi: 10.3390/jcm15176837 (PMC13566127; doi:10.3390/jcm15176837)
Supplement: Supplementary file 1 [file jcm-15-06837-s001.zip › jcm-4378141-supplementary.pdf]

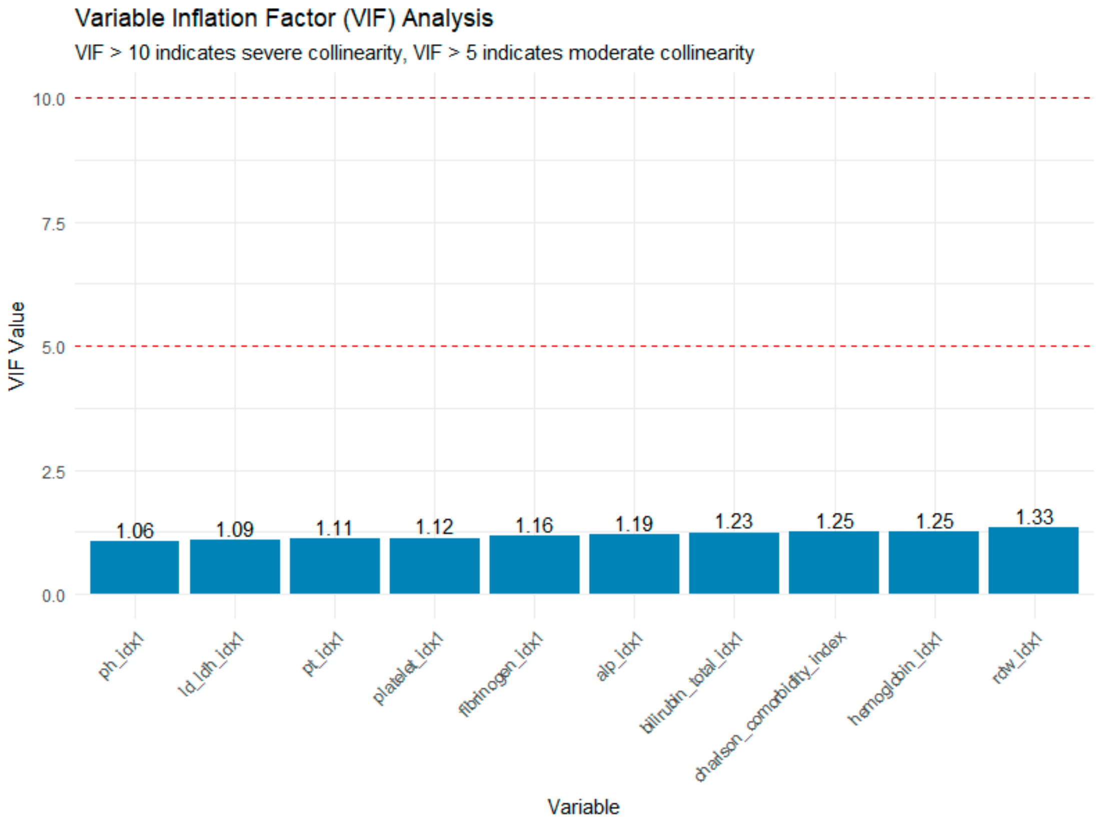

Supplementary Figure S1. the Variable Inflation Factor (VIF) analysis results

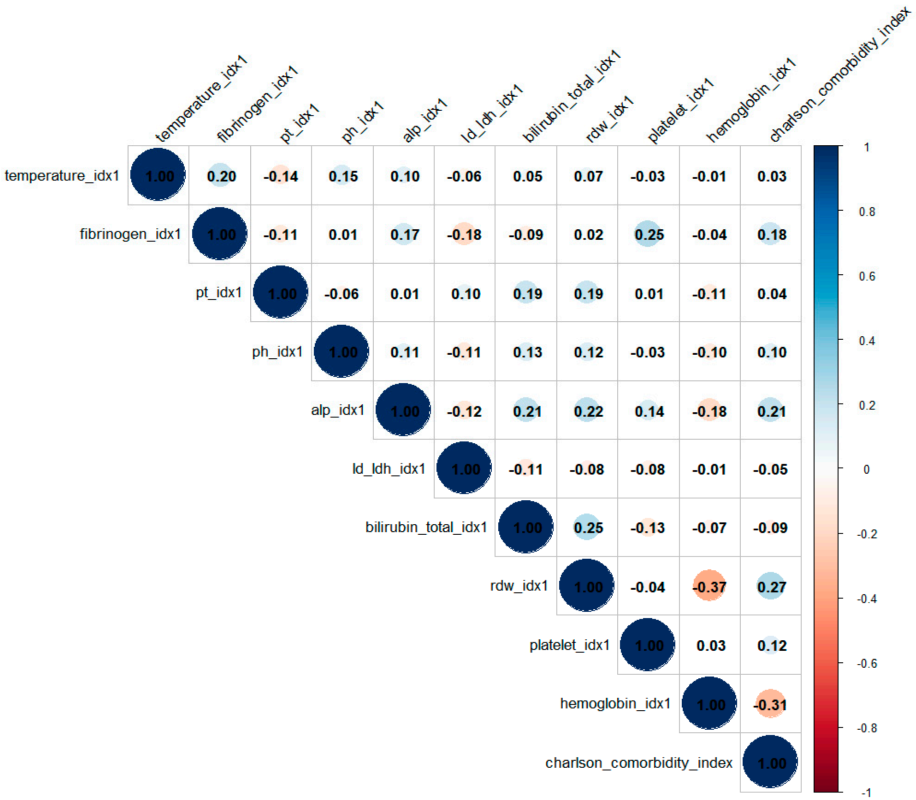

Supplementary Figure S2. Correlation cefficient matrix

Supplementary Table S1 Missing data summary

	Missing count (n)	Missing rate (%)
Age	0	0
Gender	0	0
Ethnicity	0	0
Heart Rate	0	0
Repatriation Rate	0	0
Temperature	37	7%
Mean Arterial Pressure	0	0
Fibrinogen	127	24.2%
PT	0	0
PTT	0	0
INR	0	0
Albumin	38	7.2%
PH	0	0
Base Excess	0	0
Lactate	0	0
Aniongap	0	0
BUN(mg/dL)	0	0
Sodium	0	0
Glucose	0	0
Creatinine	0	0
LDH	31	5.9%
ALT	0	0
ALP	0	0

AST	0	0
Bilirubin Total	1	0.1%
White Blood Cells	0	0
RDW	0	0
Red Blood Cells	0	0
Platelet	0	0
Hemoglobin	0	0
APS III Max	0	0
LODS Max	0	0
SOFA Max	0	0
SAPS II Max	0	0
Charlson Comorbidity	0	0
Hosp Outcome 30d(%)	0	0
LOS Icu	0	0
Hosp Time 30d	0	0

Supplementary Table S2 Fit statistics of LCTMs from 1 to 7 subclasses

Model	Number of Class	Loglik	AIC	BIC	SABIC	Entropy	% Class 1	% Class 2	% Class 3	% Class 4	% Class 5	% Class 6	% Class 7
LCTM-1	1	-5748.35	11510.69	11540.52	11518.30	1.0000	100.0						
LCTM-2	2	-5411.48	10846.95	10898.09	10860.00	0.8109	61.64	38.36					
LCTM-3	3	-5147.60	10329.20	10401.64	10347.68	0.9140	66.22	23.85	9.92				
LCTM-4	4	-5045.47	10134.95	10228.70	10158.87	0.9153	63.17	17.37	7.06	12.40			
LCTM-5	5	-5000.92	10055.84	10170.90	10085.19	0.8994	62.60	17.37	7.06	4.01	8.97		
LCTM-6	6	-4944.50	9953.00	10089.37	9987.79	0.8463	38.93	26.15	15.27	7.06	3.05	9.54	
LCTM-7	7	-4909.16	9892.31	10049.99	9932.54	0.8571	38.93	6.11	25.38	15.46	5.73	3.05	5.34

Loglik: Log-likelihood; AIC: Akaike information criterion; BIC: Bayesian information criterion; SABIC: Sample-adjusted Bayesian information criterion.

Supplementary Table S3 Mean Posterior Probability of Membership in Class in MIMIC-IV

Class	prob1	prob2	prob3	prob4	prob5	prob6	prob7
**1**	**0.9038**	0.0026	0.0923	0.0004	0.0001	0.0000	0.0008
**2**	0.0018	**0.8655**	0.0572	0.0032	0.0000	0.0162	0.0562
**3**	0.0763	0.0227	**0.8538**	0.0375	0.0002	0.0000	0.0095
**4**	0.0000	0.0032	0.0479	**0.9096**	0.0188	0.0000	0.0206
**5**	0.0000	0.0002	0.0003	0.0294	**0.9624**	0.0000	0.0078
**6**	0.0000	0.0351	0.0001	0.0000	0.0000	**0.9509**	0.0139
**7**	0.0000	0.0187	0.0000	0.0068	0.0273	0.0018	**0.9453**

Supplementary Table S4 Clinical characteristics between Group 0 and Group 1

Variables	Total (n = 524)	Group 0 (n=466)	Group 1 (n = 58)	P value
Gender, n(%)				0.11
Female	203 (38.74)	175 (37.55)	28 (48.28)	
Male	321 (61.26)	291 (62.45)	30 (51.72)	
Age, Mean ± SD	62.22 ± 16.77	62.00 ± 16.82	63.93 ± 16.44	0.41
Weight, Mean ± SD	86.80 ± 24.89	87.49 ± 25.40	81.31 ± 19.58	0.07
Height , Mean ± SD	170.02 ± 9.85	170.28 ± 9.87	167.98 ± 9.52	0.09
Los Hospital,M (Q_1, Q_3)	18.19 (9.87, 29.15)	19.11 (10.81, 29.08)	9.22 (4.94, 30.21)	**<0.01**
Los Icu, M (Q_1, Q_3)	9.80 (5.77, 15.95)	10.07 (6.04, 16.06)	6.75 (3.90, 11.14)	**<0.01**
Sofa Score, M (Q_1, Q_3)	5.00 (3.00, 7.00)	5.00 (3.00, 7.00)	5.00 (3.25, 8.00)	0.14
Hosp Time 30d, M (Q_1, Q_3)	30.00 (10.42, 30.00)	30.00 (13.58, 30.00)	8.92 (4.20, 30.00)	**<0.01**
Hosp Time 60d, M (Q_1, Q_3)	60.00 (10.42, 60.00)	60.00 (13.58, 60.00)	8.92 (4.20, 60.00)	**<0.01**
Hosp Time 90d, M (Q_1, Q_3)	90.00 (10.42, 90.00)	90.00 (13.58, 90.00)	8.92 (4.20, 90.00)	**<0.01**
Heart Rate, M (Q_1, Q_3)	98.50 (82.00,	99.00 (82.00,	94.00 (84.25,	0.96

	113.00)	112.75)	114.00)	
Map , M (Q_1, Q_3)	78.00 (69.00, 91.00)	78.00 (69.00, 91.00)	76.50 (66.25, 89.50)	0.69
Resp Rate , M (Q_1, Q_3)	21.00 (17.00, 26.00)	21.00 (16.12, 26.00)	22.00 (19.00, 27.00)	0.21
Temperature, M (Q_1, Q_3)	36.50 (36.06, 37.00)	36.56 (36.07, 37.00)	36.44 (35.71, 36.72)	**0.03**
Fibrinogen, M (Q_1, Q_3)	307.68 (187.75, 418.22)	314.71 (199.00, 432.93)	214.50 (153.25, 326.50)	**<0.01**
PT, M (Q_1, Q_3)	17.40 (14.30, 22.22)	17.10 (14.10, 21.60)	20.70 (16.40, 30.07)	**<0.01**
PTT , M (Q_1, Q_3)	35.50 (29.70, 46.92)	35.35 (29.70, 46.58)	36.65 (30.80, 50.62)	0.38
INR , M (Q_1, Q_3)	1.60 (1.30, 2.02)	1.60 (1.30, 2.00)	1.90 (1.50, 2.77)	**<0.01**
Albumin, M (Q_1, Q_3)	2.70 (2.30, 3.10)	2.70 (2.30, 3.10)	2.90 (2.30, 3.30)	0.38
PH , M (Q_1, Q_3)	7.29 (7.20, 7.37)	7.29 (7.20, 7.37)	7.23 (7.09, 7.34)	**<0.01**
Baseexcess, M (Q_1, Q_3)	-6.00 (-10.00, -2.00)	-5.50 (-10.00, -2.00)	-12.50 (-17.00, -6.00)	**<0.01**
Calcium , M (Q_1, Q_3)	8.00 (7.30, 8.50)	8.00 (7.30, 8.50)	8.05 (7.43, 8.85)	0.12
BUN , M (Q_1, Q_3)	31.00 (19.00, 49.00)	31.00 (19.00, 48.75)	35.50 (21.00, 49.75)	0.38
Potassium , M (Q_1, Q_3)	4.50 (3.90, 5.20)	4.50 (3.90, 5.18)	4.60 (4.03, 5.38)	0.22

Sodium, M (Q_1, Q_3)	138.00 (134.00, 142.00)	138.00 (134.00, 142.00)	140.00 (135.25, 141.75)	0.17
Glucose, M (Q_1, Q_3)	151.50 (114.75, 217.00)	153.50 (116.00, 217.75)	138.00 (101.75, 211.75)	0.22
Creatinine, M (Q_1, Q_3)	1.60 (1.10, 2.80)	1.60 (1.10, 2.70)	1.90 (1.22, 3.27)	0.06
ALT, M (Q_1, Q_3)	337.00 (132.00, 856.00)	334.00 (131.25, 838.00)	350.00 (146.25, 1157.75)	0.44
ALP, M (Q_1, Q_3)	116.00 (70.00, 226.50)	112.50 (68.25, 219.25)	162.00 (89.25, 261.00)	**<0.01**
AST, M (Q_1, Q_3)	483.00 (174.25, 1476.50)	465.00 (160.25, 1406.00)	588.50 (240.00, 1763.00)	0.22
LDH, M (Q_1, Q_3)	806.00 (451.00, 1958.75)	753.00 (425.25, 1797.75)	1410.50 (700.00, 3152.50)	**<0.01**
Bilirubin Total, M (Q_1, Q_3)	1.30 (0.70, 2.92)	1.20 (0.60, 2.70)	1.50 (0.90, 3.70)	**0.03**
white blood cells , M (Q_1, Q_3)	14.30 (9.88, 20.22)	14.25 (10.10, 19.90)	15.15 (8.45, 23.50)	0.50
Lymphocytes, M (Q_1, Q_3)	1.02 (0.61, 1.47)	1.03 (0.64, 1.45)	0.98 (0.53, 1.77)	0.75
RDW, M (Q_1, Q_3)	15.10 (13.90, 16.90)	14.90 (13.80, 16.80)	16.00 (14.80, 18.60)	**<0.01**
Red Blood Cells , M (Q_1, Q_3)	3.59 (3.00, 4.22)	3.66 (3.02, 4.25)	3.29 (2.77, 3.83)	**0.01**
Platelet , M (Q_1, Q_3)	167.00 (110.00,	170.50 (115.00,	141.50 (55.75,	**0.03**

	249.00)	251.75)	218.25)	
Hemoglobin, M (Q_1, Q_3)	10.70 (8.90, 12.70)	10.80 (9.12, 12.88)	9.70 (7.93, 11.50)	**<0.01**
Charlson Comorbidity, M (Q_1, Q_3)	5.00 (3.00, 8.00)	5.00 (3.00, 7.00)	6.00 (4.00, 9.00)	**0.02**

Group 0: included class 1, 2, 3, 4 and 6

Group 1:included class 5 and 7

Q1, 1st Quartile; Q3, 3st Quartile.

MAP, mean arterial pressure; PT, prothrombin time; INR, international normalisation ratio; PTT, partial thromboplastin time; BUN, blood urea nitrogen; ALT, alanine aminotransferase; AST, aspartate aminotransferase; ALP,Alkaline Phosphatase; LDH, lactate dehydrogenase; RDW, red blood cell distribution width;
